# Aldose reductase deficiency inhibits LPS-induced M1 response in macrophages by activating autophagy

**DOI:** 10.1186/s13578-021-00576-7

**Published:** 2021-03-26

**Authors:** Peng Cheng, Jianwei Xie, Zhiyong Liu, Jian Wang

**Affiliations:** 1 Institute of Neurosciences, Fourth Military Medical University, Xi’an, 710032 China; 2 Department of Neurology, 425th Hospital of the People’s Liberation Army, Sanya, 572000 China

**Keywords:** Aldose reductase, Bone marrow-derived macrophages, Macrophage polarization, Autophagy, Ubiquitination, Nuclear factor κB

## Abstract

Macrophage M1 polarization mediates inflammatory responses and tissue damage. Recently, aldose reductase (AR) has been shown to play a critical role in M1 polarization in macrophages. However, the underlying mechanisms are unknown. Here, we demonstrated, for the first time, that AR deficiency repressed the induction of inducible nitric oxide synthase in lipopolysaccharide (LPS)-stimulated macrophages via activation of autophagy. This suppression was related to a defect in the inhibitor of nuclear factor κB (NF-κB) kinase (IKK) complex in the classical NF-κB pathway. However, the mRNA levels of IKKβ and IKKγ were not reduced in LPS-treated AR knockout (KO) macrophages, indicating that their proteins were downregulated at the post-transcriptional level. We discovered that LPS stimuli induced the recruitment of more beclin1 and increased autophagosome formation in AR-deficient macrophages. Blocking autophagy through 3-methyladenine and ammonium chloride treatment restored IKKβ and IKKγ protein levels and increased nitric oxide synthase production in LPS-stimulated AR-deficient macrophages. More assembled IKKβ and IKKγ underwent ubiquitination and recruited the autophagic adaptor p62 in LPS-induced AR KO macrophages, promoting their delivery to autophagosomes and lysosomes. Collectively, these findings suggest that AR deficiency is involved in the regulation of NF-κB signaling, and extends the role of selective autophagy in fine-tuned M1 macrophage polarization.

**Supplementary Information:**

The online version contains supplementary material available at 10.1186/s13578-021-00576-7.

## Introduction

Macrophages demonstrate significant plasticity and can modify their phenotype and function in response to their microenvironment [1]. Macrophages are roughly categorized into two different subsets, namely, inflammatory M1 and anti-inflammatory M2 macrophages [2]. In response to lipopolysaccharide (LPS) treatment, either alone or in combination with pro-inflammatory cytokines such as interferon-γ, macrophages undergo M1 polarization, characterized by the expression of pro-inflammatory cytokines and cytotoxic mediators (reactive oxygen and nitrogen species), as well as increased phagocytic and antigen-presenting capacity [3].

Aldose reductase (AR), a rate-limiting enzyme in the polyol pathway that catalyzes the reduction of glucose to sorbitol in the presence of reduced nicotinamide adenine dinucleotide phosphate, has emerged as a molecular target in multiple inflammatory diseases [4]. Ravindranath et al. demonstrated that transgenic mice overexpressing AR showed a more pronounced inflammatory response in a cecal ligation and puncture model [5]. In addition, AR inhibition suppresses inflammatory disorders or immune responses in several other animal models [6–10]. It was reported that M1-polarized human monocyte-derived macrophages expressed significantly higher levels of AR mRNA and AR protein compared with M2-polarized macrophages in vitro [11]. Accumulating evidence implicates the classical nuclear factor-κB (NF-κB) signaling pathway in the modulation of M1 macrophage polarization [12–15]. Previously, Zhang et al. reported that the expression of AR is upregulated after spinal cord injury in wild type (WT) mice while phosphorylated NF-κB is downregulated, and that the number of M1-like macrophages is decreased after spinal cord injury in AR knockout (KO) mice [16]. However, the exact mechanisms involved are unknown.

Recently, it has been reported that AR deficiency or inhibition enhances autophagy in mouse cardiac myocytes under pathological cardiac hypertrophy or fasting conditions [17, 18]. Autophagy is an essential cell-intrinsic mechanism that affords protection against starvation. Moreover, it represents a quality control system that can deliver damaged organelles, worn-out or misfolded proteins, and invading microorganisms from the cytoplasm to the lysosomes for degradation [19]. Defects in autophagy are linked to many human diseases and to the function of cells of the immune response [20]. Experimentally, Toll-like receptor 4 (TLR4) is a sensor for autophagy associated with innate immunity in macrophages [21].

Herein, we demonstrate that LPS treatment leads to a defect at the IKK complex level and the production of more autophagosomes in AR-deficient macrophages. These findings prompted us to investigate the inherent relationships between these processes.

## Materials and methods

### Isolation and culture of BMMs

**Fig. 1:**
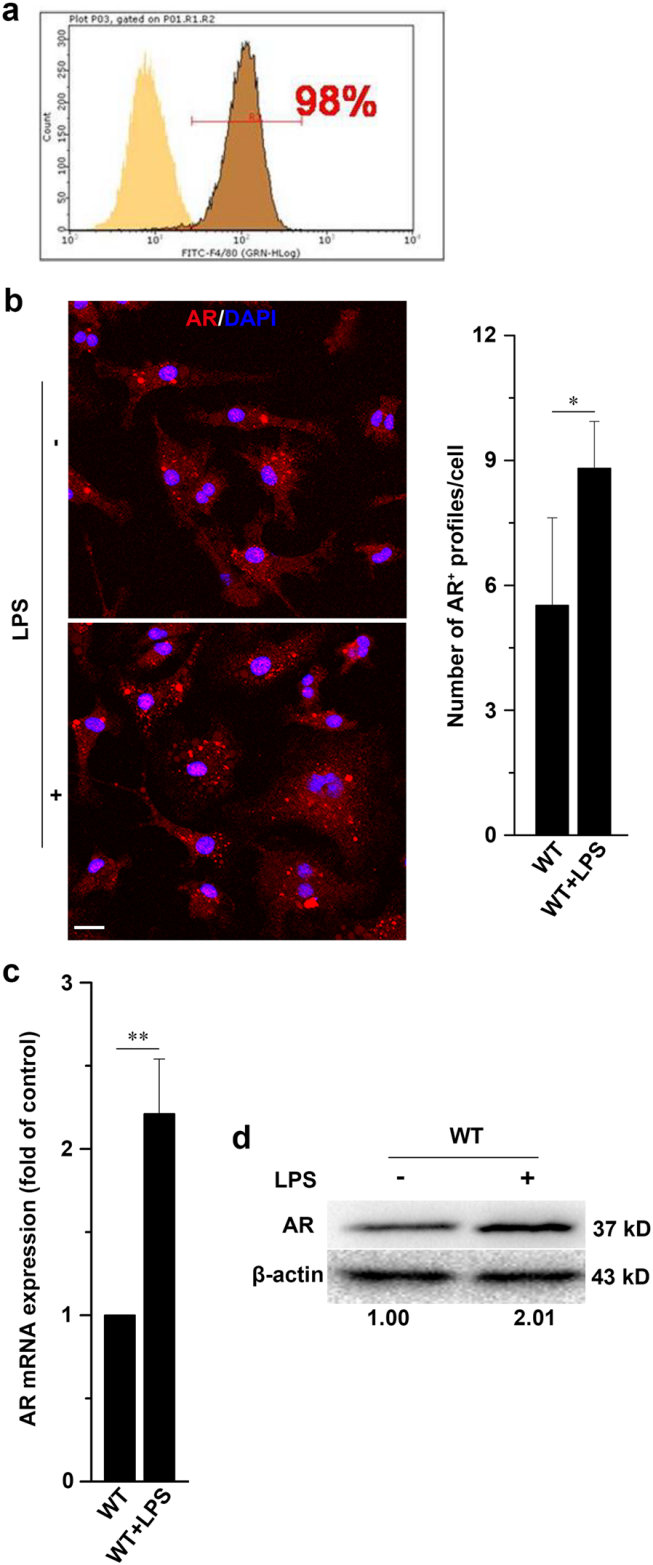
Identification of BMMs in vitro and LPS stimulation upregulates AR expression in BMMs. **a** Following induction of BMMs from the bone marrow, approximately 98% of the cells were F4/80-positive. **b** Confocal microscopy of BMMs from WT mice left untreated or treated for 24 h with LPS (500 ng/ml), and then immunostained for AR. Scale bars = 10 µm. Right graph shows quantification analysis of the number of AR + foci per cell. **c**, **d** mRNA and protein levels of AR in BMMs from WT mice left untreated or treated with 500 ng/ml LPS for 24 h. Transcription levels are expressed as fold change of control and densitometric AR/β-actin ratios are shown below the blot. Data are expressed as the mean ± SD from three independent experiments, *P < 0.05, **P < 0.01

WT and AR KO C57BL/6 J mice were bred at the Laboratory Animal Center of Fourth Military Medical University. Bone marrow cells were isolated from adult mice and processed as described previously [22]. Then, the cells were cultured in DMEM (Gibco, Carlsbad, CA, USA) supplemented with 0.001% β-mercaptoethanol, 1% penicillin/streptomycin, 1% HEPES, 10% FBS (Gibco), and 20% sL929 supernatant obtained from sL929 cells, which secrete macrophage colony-stimulating factor (M-CSF) required for the promotion of hematopoietic stem cell differentiation into macrophages. After 7 days of incubation with the conditioned medium, the floating cells were removed, and the viable cells attached to flat-bottomed 6- or 24-well plastic culture plates or 75 cm^2^ culture flasks (Nunc, Roskilde, Denmark) were used to obtain BMMs. Next, the spent culture media were changed to complete medium without sL929 cell culture supernatant, then the cells were further cultured for 3 days to restore the BMMs to their resting state. Flow cytometry analysis of surface antigens showed that 98% of the cultured cells expressed F4/80, a specific marker of macrophages (Fig. 1a).

### Reagents

LPS (*E. coli* 0111:B4), ammonium chloride (NH_4_Cl), and 3-methyladenine (3-MA) were purchased from Sigma-Aldrich (St. Louis, MO, USA). The reagents were used at the indicated concentrations.

### Flow cytometry

BMMs were incubated in blocking solution (rat serum, 20 min at 4 ℃) and were subsequently stained with FITC-conjugated anti-F4/80 (1:100, AbD Serotec, Hercules, CA, USA) in the dark for 20 min at 4 °C. Acquisitions were performed on a Millipore flow cytometer (Guawa 6HT). Subsequent data analyses were completed using FlowJo software version 7.6.2 (Tree Star, Ashland, OR, USA). Results are expressed as % of positive cells.

### Immunofluorescence

Cells were cultured to ~ 70–80% confluence using glass coverslips in 24-well plates. After treatment, primary BMMs were fixed with 4% paraformaldehyde in phosphate-buffered saline (PBS, pH = 7.4) for 30 min and then washed thrice with PBS. Non-specific antibody binding was blocked by incubating the cells for 1 h at room temperature (20–25 °C) in PBS containing 5% bovine serum albumin and 0.3% Triton X-100. The cells were then stained with the following primary antibodies overnight at 4 °C: mouse anti-AR (1:200, Santa Cruz Biotechnology, Santa Cruz, CA, USA), mouse anti-beclin1 (1:200, Santa Cruz Biotechnology), goat anti-microtubule-associated protein 1 light chain 3β (LC3β, 1:200, Santa Cruz Biotechnology), mouse anti-lysosome-associated membrane protein 1 (LAMP1, 1:200, Santa Cruz Biotechnology), rabbit anti-IKKβ (1:100, Epitomics, Burlingame, CA, USA), rabbit anti-IKKγ (1:200, Santa Cruz Biotechnology,), goat anti-p62 (1:200, Santa Cruz Biotechnology), and mouse anti-ubiquitin specific for K63 (PloyUb, 1:400, Abcam, Cambridge, UK). After washing with PBS, the cells were incubated for 1 h at room temperature in the dark with their respective secondary antibodies: fluorescein isothiocyanate-, DyLight 488-, and DyLight 594-conjugated secondary antibodies (1:500 ~ 800, Jackson ImmunoResearch Laboratories, West Grove, PA, USA). Diamidinophenylindole (DAPI, 1 μg/ml, Sigma-Aldrich) was applied to visualize the nuclei. Rhodamine-phalloidin (1:250, Life Technologies-Invitrogen, Carlsbad, USA) was used to stain the actin cytoskeleton of the cells. The cells were observed using a laser confocal microscope (FluoView FV1000 MPE, Olympus Corporation, Tokyo, Japan). We applied the JACoP plugin of Image J (National Institutes Health, Bethesda, MD, USA) to perform co-localization analysis of any image pair, and the co-localization rate of the green and red signals was evaluated using Manders’ overlap coefficient [23].

### Western blotting

The extraction of cellular proteins and their detection via western blotting were performed as previously described [21, 24]. Western blotting was performed using the following primary antibodies: mouse anti-AR, mouse anti-beclin1, goat anti-LC3β, mouse anti-LAMP1, and rabbit anti-IKKγ antibodies (1:500, Santa Cruz Biotechnology); mouse anti-TLR4, rabbit anti-iNOS, rabbit anti-IKKα, and rabbit anti-4-HNE (1:1000, Abcam); rabbit anti-IKKβ and rabbit anti-phospho-IκBα antibodies (1:1000, Epitomics); rabbit anti-phospho-IKKα/β, rabbit anti-IκBα, rabbit anti-p65, and rabbit anti-phospho-p65 antibodies (1:1000, Cell Signaling Technology, Danvers, MA, USA); and mouse anti-β-actin antibodies (1:8000, Sigma-Aldrich). The blots were then incubated with their respective secondary antibodies: horseradish peroxidase-conjugated goat anti-rabbit IgG, goat anti-mouse IgG (both 1:8000, Abcam), and donkey anti-goat IgG (1:5000, Santa Cruz Biotechnology). β-actin was used as the loading control. The immunoreactive bands were scanned using the Bio-Rad ChemiDoc™ XRS^+^ imager with Image Lab™ Software (Bio-Rad Laboratories, CA, USA). Band intensity was quantified using Quantity One software (Bio-Rad Laboratories).

### Quantitative reverse transcription-polymerase chain reaction (qRT-PCR)

Total RNA from BMMs was isolated using TRIzol Reagent (Invitrogen, Carlsbad, CA, USA) according to the manufacturer’s instructions. PCR was performed on a Bio-Rad CFX 96™ Real-Time system (Bio-Rad Laboratories) employing a SYBR Green qPCR core reagent kit (DRR081A, Takara Bio Inc., Otsu, Japan). qRT-PCR was performed using the following primers, specific for mouse AR: forward primer, 5′-ACGGCTATGGAACAACTA-3′ and reverse primer, 5′-TGTGGCAGTATTCAATCAG-3′; mouse IKKβ: forward primer, 5′-GGAGCCTGGGAAATGAAAGAA-3′ and reverse primer, 5′-GCCAGAGCCCTACCTGATTG-3′; mouse IKKγ: forward primer, 5′-AAGCACCCCTGGAAGAACC-3′ and reverse primer, 5′-CCTGCTCTGAAGGCAGATGTA-3′; mouse iNOS: forward primer, 5′-CCCTTCAATGGTTGGTACATGG-3′ and reverse primer, 5′- ACATTGATCTCCGTGACAGCC-3′; mouse CD86: forward primer, 5′-TTGTGTGTGTTCTGGAAACGGAG-3′ and reverse primer, 5′- AACTTAGAGGCTGTGTTGCTGG-3′; and mouse β-actin: forward primer, 5′-CGTGCGTGACATCAAAGAGAA-3′ and reverse primer, 5′-GCTCGTTGCCAATAGTGATGA-3′. Gene expression was normalized using β-actin as an internal control, and fold changes were calculated.

### Transmission electron microscopy

Cells were grown to ~ 80% confluence in 75 cm^2^ culture flasks. After stimulation, BMMs were harvested and centrifuged at 1500×*g* for 10 min. The pellets were fixed in a mixture of 4% paraformaldehyde and 0.05% glutaraldehyde, post-fixed in 0.5% osmium tetroxide, dehydrated in a graded ethanol series and propylene oxide, and embedded in epoxy resin. Ultrathin sections were obtained using an ultramicrotome (EM UC6, Leica Microsystems, Baden-Württemberg, Germany), mounted on mesh grids (6–8 sections/grid), and counter-stained with uranyl acetate and lead citrate. The sections were viewed using a transmission electron microscope (JEM-1230, JEOL, Tokyo, Japan) equipped with a Gatan digital camera.

### Statistical analysis

All data are expressed as the mean ± SD. Statistically significant differences between the mean values were determined using two-tailed Student’s t-test. Differences at *P* < 0.05 were considered statistically significant. *P < 0.05; **P < 0.01.

## Results

### LPS stimulation upregulates AR expression in BMMs

It has been reported that AR can regulate inflammatory signals and immune responses in several animal disease models [5–8]. Since inflammatory signals and immune responses are tightly correlated with macrophage polarization, we investigated changes in the expression of AR upon LPS stimulation in BMMs from WT C57BL/6 mice.

We used immunofluorescence staining to label AR in BMMs. The number of AR-immunoreactive profiles in BMMs was significantly increased after treatment with LPS for 24 h (Fig. 1b). We next used qRT-PCR and western blotting to detect the changes of AR mRNA and protein in LPS-stimulated BMMs. The transcription and expression of AR were both significantly increased following treatment with LPS for 24 h (Fig. 1c, d). Collectively, these results indicate that AR expression is triggered upon stimulation of BMMs with LPS.

### AR deficiency suppresses the M1 response and NF-κB activation at the IKK complex level in LPS-stimulated BMMs

In response to LPS treatment, macrophages undergo M1 polarization, characterized by the expression of inducible nitric oxide synthase (iNOS) [25]. Activation of the canonical NF-κB signaling pathway in response to LPS was demonstrated by monitoring iNOS expression, immunoblot analysis, and the measurement of nitric oxide (NO) production—important downstream products of LPS signaling in macrophages [26].

**Fig. 2:**
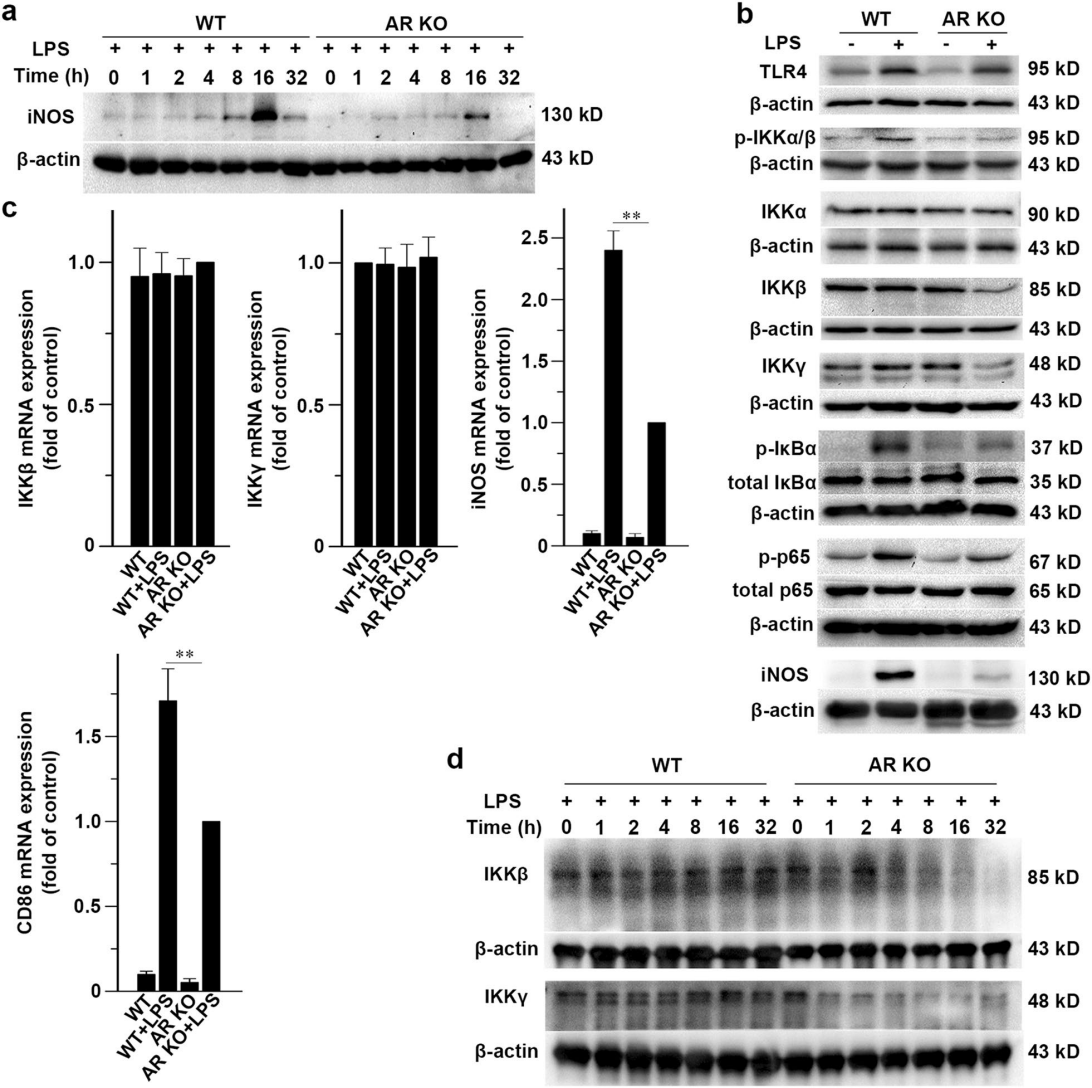
Stimulation of AR-deficient macrophages with LPS decreases the protein levels of IKKβ and IKKγ. **a** BMMs from WT or AR KO mice were treated with 500 ng/ml LPS for the indicated time periods, and the production of iNOS was assayed. **b** Effect of AR deficiency on several important components of the TLR4/NF-κB signaling pathway. Immunoblot analysis for TLR4, p-IKKα/β, IKKα, IKKβ, IKKγ, IκBα, p-IκBα, p65, p-p65, and iNOS in BMMs from WT or AR KO mice treated with or without 500 ng/ml LPS for 16 h. **c** qRT-PCR of IKKβ, IKKγ, iNOS, and CD86 mRNA in BMMs from WT or AR KO mice treated with or without 500 ng/ml LPS for 16 h. Transcription levels are expressed as fold change of control. **d** BMMs from WT or AR KO mice were treated with 500 ng/ml LPS for the indicated time periods. Data represent the mean ± SD of three different experiments

We hypothesized the existence of a relationship between AR and the NF-κB p65 signaling pathway. To test this hypothesis, we carried out experiments on BMMs from WT or AR KO mice. Stimulation of BMMs with 500 ng/ml LPS increased the levels of iNOS in a time-dependent manner during the first 32 h (Fig. 2a). Expression of iNOS peaked at 16 h after LPS treatment (Fig. 2a). The induction of iNOS upon LPS stimulation decreased markedly when the AR gene was knocked out (Fig. 2a). These results suggested that the LPS-stimulated induction of iNOS was closely associated with AR.

For most inducers of the classical NF-κB signaling pathway, IKKα is not required for the phosphorylation of IκBα; however, genetic studies have shown that NF-κB p65 signaling requires IKKβ and IKKγ to phosphorylate IκBα [27]. The canonical NF-κB pathway has several important components, including TLR4, IKKβ, IKKγ, IκBα, and p65 [28–30]. Following 16 h of LPS exposure, we detected lower levels of phospho-IKKα/β (p-IKKα/β), IKKβ, IKKγ, phospho-IκBα (p-IκBα), phospho-p65 (p-p65), and iNOS proteins in AR KO BMMs than in control cells (Fig. 2b). We used qRT-PCR to measure the mRNA levels of IKKβ, IKKγ, iNOS, and CD68 in the total RNA obtained from the BMMs after 16 h of treatment with or without LPS. The results showed that mRNA levels of IKKβ and IKKγ were not affected in the LPS-treated AR KO BMMs (Fig. 2c). In AR KO macrophages, the mRNA levels of iNOS were less increased compared to that in WT cells after LPS stimulation (Fig. 2c). The mRNA level of CD86, another M1 marker, was also less increased in AR KO BMMS compared to that in WT cells after LPS treatment (Fig. 2c). The above data indicated that the suppression of the M1 response in AR-deficient macrophages after LPS stimulation was due to the post-transcriptional degradation of IKKβ and IKKγ. To determine the temporal patterns of change in the protein levels of IKKβ and IKKγ, we stimulated BMMs from WT or AR KO mice with LPS for 32 h and immunoblotted the lysates for IKKβ and IKKγ. The time-course experiments revealed that LPS induced a gradual reduction of IKKβ and IKKγ proteins in BMMs from AR KO mice (Fig. 2d).

### AR deficiency enhances autophagosome formation and maturation in LPS-stimulated BMMs

**Fig. 3:**
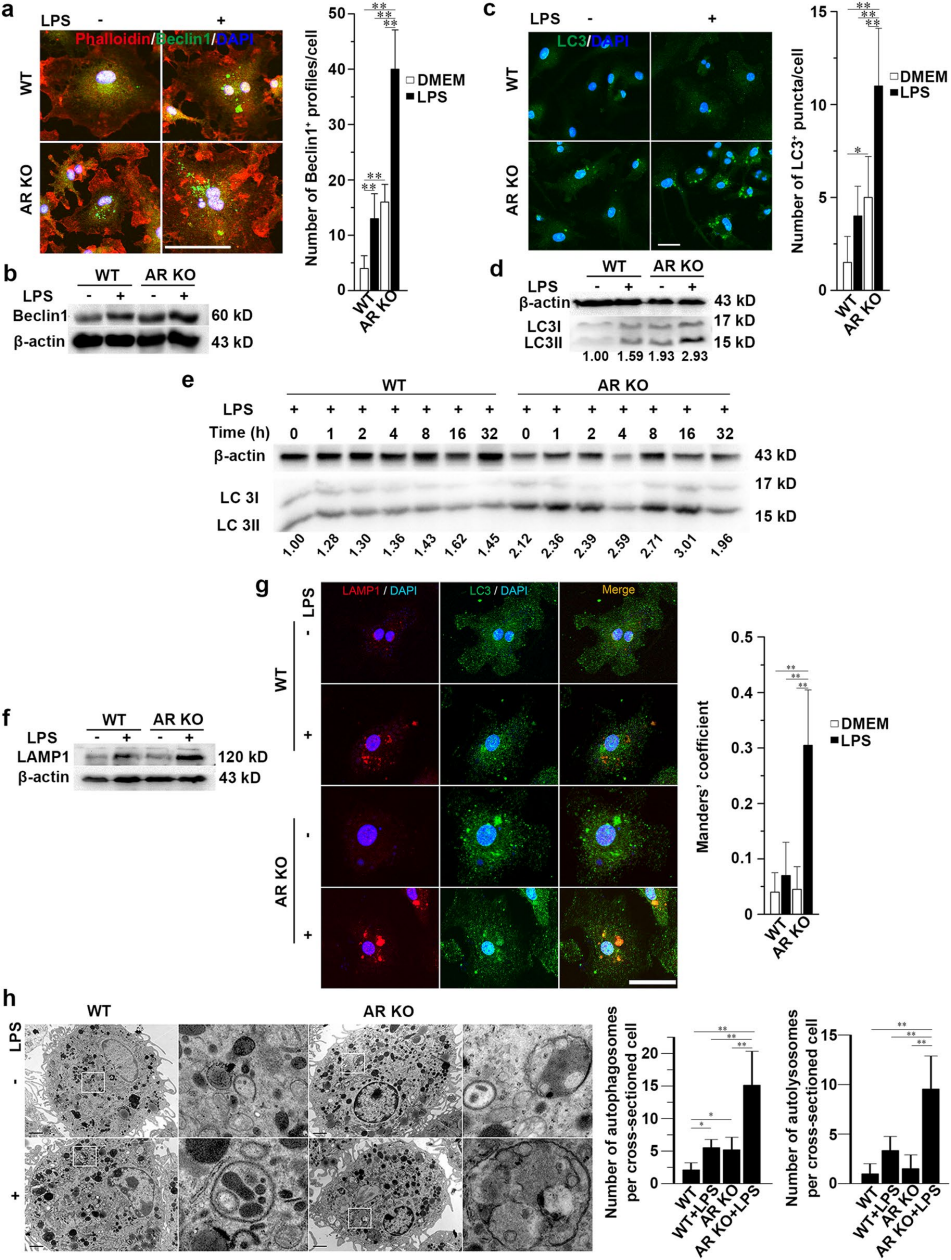
AR deficiency enhances autophagosome formation and maturation in LPS-stimulated macrophages. **a** BMMs from WT or AR KO mice were treated with or without 500 ng/ml LPS for 16 h and then immunostained for beclin1. Right graph represents quantification analysis of the number of beclin1 + foci per cell. Scale bars = 50 µm. **b** Immunoblot analysis of beclin1 in BMMs from WT or AR KO mice treated with or without 500 ng/ml LPS for 16 h. **c** Induction of LC3 + vacuoles in BMMs from WT or AR KO mice treated with or without 500 ng/ml LPS for 16 h and then immunostained for LC3. Right graph represents the quantification analysis of the number of LC3 + puncta per cell. Scale bars = 20 µm. **d** Immunoblot analysis of LC3 in BMMs from WT or AR KO mice treated with or without 500 ng/ml LPS for 16 h. Densitometric LC3II/LC3I/β-actin ratios are shown below the blot. **e** Time-course analysis of autophagy in macrophages during the 32 h of LPS stimulation. BMMs from WT or AR KO mice were treated with 500 ng/ml LPS for the indicated durations. Densitometric LC3II/LC3I/β-actin ratios are shown below the blot. **f** Immunoblot analysis of LAMP1 in BMMs from WT or AR KO mice treated with or without LPS for 16 h. **g** Confocal microscopy of BMMs from WT or AR KO mice treated with or without 500 ng/ml LPS for 16 h and immunostained for LC3 and LAMP1. Scale bars = 20 µm. Right graphs represent co-localization of LAMP1 and LC3 signals evaluated using Manders’ overlap coefficient. **h** Ultrastructural analysis of autophagy via transmission electron microscopy in BMMs. WT or AR KO BMMs were treated with or without 500 ng/ml LPS for 16 h. The panel on the right is the enlargement of the outlined area on the left. Scale bars = 1 μm. Right graphs represent the quantification of the number of autophagosomes and autolysosomes per cross-sectioned cell. Data are expressed as the mean ± SD from three independent experiments, *P < 0.05, **P < 0.01

To determine whether autophagosome formation occurred because of AR deficiency, we treated BMMs from WT or AR KO mice with or without LPS. Beclin1 is a key component of the class III phosphatidylinositol 3-kinase (PI3K) complex, which initiates autophagosome formation [31]. Incubation of BMMs with 500 ng/ml LPS for 16 h led to the recruitment of beclin1, as shown in the change in beclin1 staining from a diffuse to a punctate pattern (Fig. 3a). We then conducted a detailed quantitative analysis of LPS-stimulated beclin1 aggregation. LPS induced a significant increase in the number of beclin1^+^ profiles in AR KO cells (Fig. 3a). The increase in beclin1 levels was also confirmed through immunoblotting (Fig. 3b).

In macroautophagy, cells form double-membraned vesicles, known as autophagosomes, around a portion of the cytoplasm [32]. Two forms of LC3, LC3I, and LC3II are produced post-transcriptionally; LC3I is cytosolic, whereas LC3II is membrane-bound and is mainly enriched in the autophagosomal vacuoles, and the amount of LC3II is correlated with the extent of the autophagosome formation [33, 34]. The autophagic vacuoles can be imaged using confocal microscopy via immunofluorescence staining for LC3, which delineates them in the cytoplasm [35]. We then conducted a detailed quantitative analysis of LPS-stimulated autophagy. Without LPS treatment, AR KO macrophages exhibited more LC3^+^ vacuoles compared to WT cells (Fig. 3c). After LPS induction, there was a significant increase in the number of autophagosomes in AR KO BMMs (Fig. 3c). The increase in autophagic flux was confirmed by immunoblotting with an LC3 antibody (Fig. 3d). To observe the temporal pattern of autophagy within 32 h, we immunoblotted lysates from LPS-stimulated WT or AR KO BMMs for LC3. The time-course experiment showed that LPS treatment significantly increased autophagic flux in AR KO cells compared with WT controls within 32 h (Fig. 3e).

As their maturation proceeds, autophagosomes fuse with lysosomes, resulting in the degradation of their contents [36]. LAMP1 is widely used as a lysosome marker. The merging of LC3 with LAMP1 indicates the presence of mature autophagosomes (autolysosomes). Immunoblotting showed that LAMP1 was significantly upregulated in LPS-stimulated AR KO cells (Fig. 3f). In AR KO BMMs, LPS promoted the maturation of autophagosomes as evidenced by an increase in the co-localization of LC3 with LAMP1 (Fig. 3g).

Transmission electron microscopy was also used to examine the effects of LPS on autophagy in BMMs from WT or AR KO mice. The number of double-membrane vacuoles, a typical feature of autophagosomes, was markedly increased in non-stimulated AR KO macrophages compared with that in WT cells; however, the fusion of primary lysosomes and autophagosomes, a typical feature of autolysosomes, was rare in AR KO and WT macrophages without LPS stimulation (Fig. 3h). Furthermore, it was observed that LPS treatment induced more autophagosomes and autolysosomes in AR-deficient cells compared to the controls (Fig. 3h).

Collectively, these results indicate that AR deficiency promotes autophagosome biogenesis in non-stimulated macrophages, and further strengthens biogenesis and maturation in LPS-activated BMMs.

### AR deficiency mediates autophagic degradation of IKKβ and IKKγ in LPS-treated BMMs

**Fig. 4:**
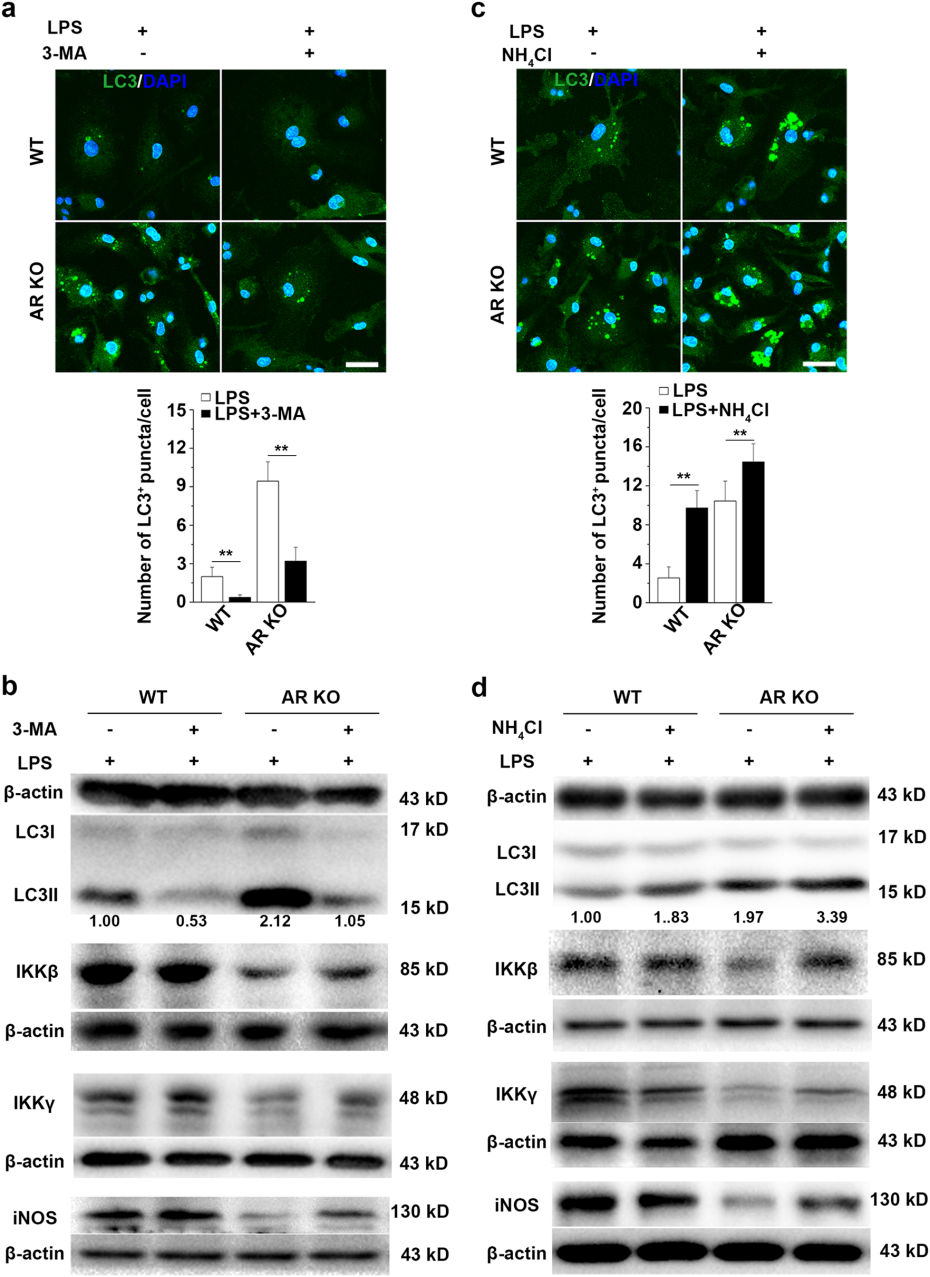
AR deficiency mediates the autophagic degradation of IKKβ and IKKγ in LPS-stimulated macrophages. **a**, **c** WT or AR KO BMMs were incubated for 16 h in the presence of LPS (500 ng/ml), LPS + 3-MA (3 mM), or LPS + NH_4_Cl (10 mM), followed by immunostaining for LC3. Lower graphs represent the quantification of the number of autophagosomes per cell. Scale bars = 20 µm. **b**, **d** Immunoblot analysis for LC3, IKKβ, IKKγ, and iNOS in lysates of WT or AR KO BMMs treated with 500 ng/ml LPS for 16 h in the presence or absence of 3-MA (3 mM) or NH_4_Cl (10 mM). Densitometric LC3II/LC3I/β-actin ratios are shown below the blot. Data are expressed as the mean ± SD from three independent experiments. *P < 0.05, **P < 0.01

The addition of the PI3K inhibitor 3-MA has been shown to block autophagosome formation [37]. Our results showed that 3-MA (3 mM) was able to block LPS-stimulated autophagy as detected by LC3 immunofluorescence (Fig. 4a) and immunoblotting (Fig. 4b). NH_4_Cl is a commonly used lysosome inhibitor. It localizes to acidic vesicles and impairs lysosomal acidification and protease activity, resulting in the accumulation of autophagosomes by impairing their fusion with lysosomes [38]. Incubation of the cells with 10 mM NH_4_Cl for 16 h resulted in the accumulation of LC3^+^ vacuoles (Fig. 4c) and a large increase in LC3II levels (Fig. 4d) in LPS-stimulated macrophages.

To investigate the mechanisms underlying the observed downregulation of key components of the IKK complex and enhanced autophagy, we analyzed the protein levels of IKKβ and IKKγ via immunoblotting in LPS-stimulated BMMs after inhibiting autophagy using 3-MA. Our results revealed that the protein levels of IKKβ and IKKγ were rescued following 3-MA administration in LPS-treated AR-deficient cells (Fig. 4c). The induction of iNOS in LPS-treated AR KO macrophages was also significantly increased after 3-MA treatment (Fig. 4c). This was further supported by our data showing that the protein levels of IKKβ and IKKγ were restored as reflected by the increased levels of iNOS in LPS-stimulated AR-deficient cells upon addition of NH_4_Cl (Fig. 4d). These results suggest that IKKβ and IKKγ were loaded into autophagosomes and subsequently degraded in lysosomes, which limited iNOS induction and M1 polarization in LPS-activated AR-deficient BMMs.

### AR deficiency facilitates IKKβ and IKKγ to co-localize with autophagosomes and lysosomes in LPS-stimulated BMMs

**Fig. 5:**
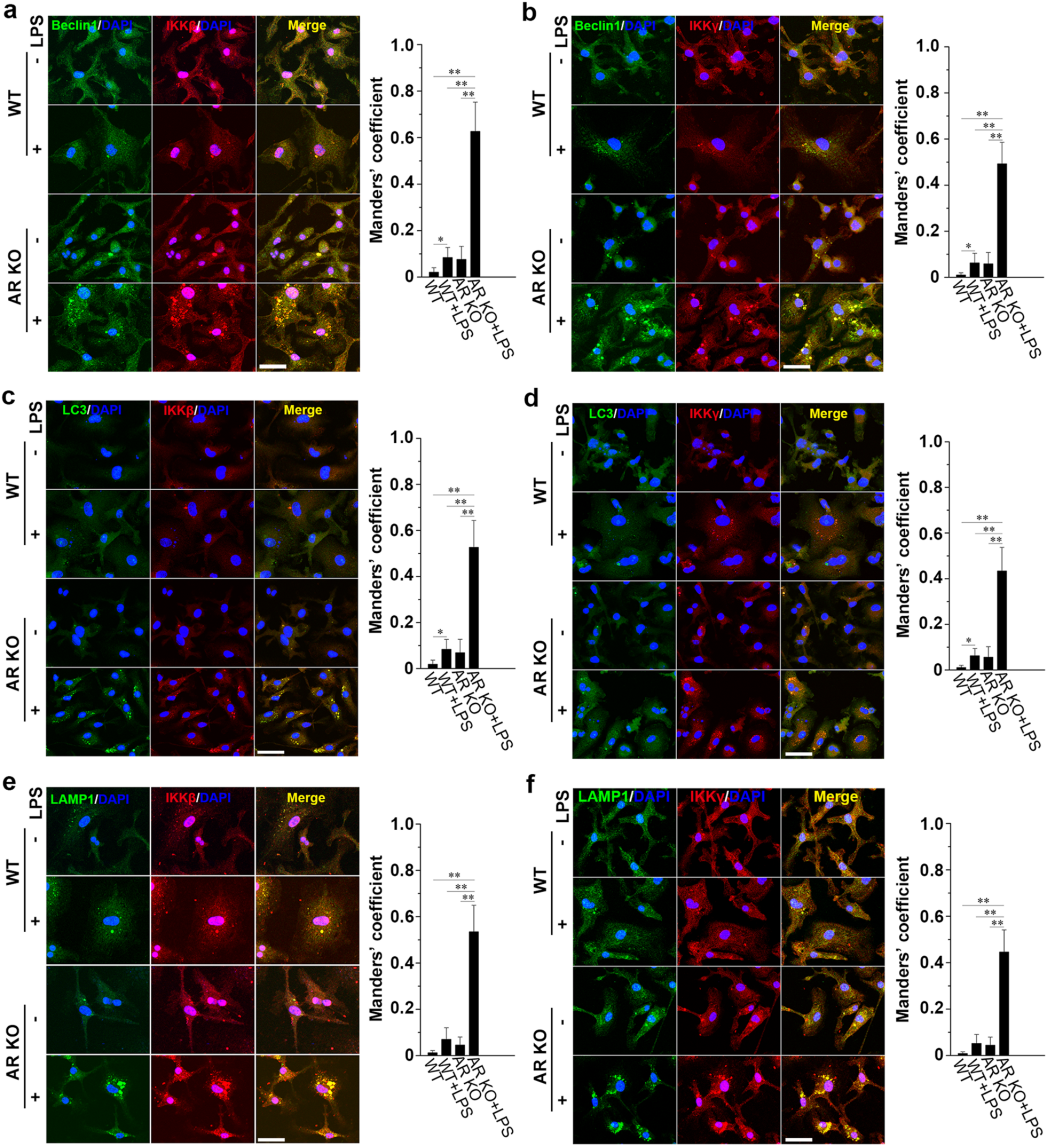
AR deficiency facilitates IKKβ and IKKγ to co-localize with autophagosomes and lysosomes in LPS-stimulated macrophages. Confocal microscopy of BMMs from WT or AR KO mice treated with or without 500 ng/ml LPS 1 h and co-stained for **a** beclin1 and IKKβ, **b** beclin1 and IKKγ, **c** LC3 and IKKβ, **d** LC3 and IKKγ, **e** LAMP1 and IKKβ, and **f** LAMP1 and IKKγ. Scale bars = 20 µm. Right graphs represent the quantification of co-localization of signals using Manders’ overlap coefficient. Data are expressed as the mean ± SD from three independent experiments, *P < 0.05, **P < 0.01

In the initial phase of autophagy, beclin1 serves as a platform to recruit autophagy activators and inhibitors [39]. We immunostained BMMs for IKKβ, IKKγ, and beclin1 after 1 h of incubation with LPS. We showed that more intracellular structures were labeled with IKKβ and IKKγ co-localized with beclin1^+^ foci in AR KO BMMs after exposure to LPS (Fig. 5a, b). These results indicate that in the initial induction of autophagy, IKKβ and IKKγ are recruited by beclin1.

To verify that IKKβ entered the autophagosomes, we performed immunocytochemistry to label IKKβ and autophagic vacuoles. We observed autophagosome formation 1 h after treatment with LPS. Autophagosome induction was poor and only a few overlapping IKKβ-labeled structures and LC3-labeled puncta were observed in resting macrophages (Fig. 5c). Upon LPS stimulation, a higher number of IKKβ^+^ structures co-localized with LC3^+^ vacuoles was observed in AR KO macrophages than in WT macrophages (Fig. 5c). Similarly, AR knockout increased LC3 staining and its co-localization with IKKγ in LPS-treated BMMs (Fig. 5d). Next, we detected higher levels of co-localization between the lysosome marker LAMP1 and IKKβ and IKKγ in LPS-induced AR KO BMMs (Fig. 5e, f). These results indicate that after recruitment by beclin1, IKKβ and IKKγ can be engulfed by autophagosomes and probably be degraded after fusion with lysosomes.

### AR deficiency, polyubiquitination, and p62 co-mediate the autophagic degradation of IKKβ and IKKγ in LPS-stimulated BMMS

Autophagic adaptors represent a mechanism through which intracellular targets are delivered to autophagosomes [40]. An adaptor protein, p62, recognizes polyubiquitinated targets and binds to the ubiquitin-like autophagosome membrane LC3 in the autophagic degradation pathway [41]. It has been shown that TLR4-mediated autophagy is a p62-dependent type of selective autophagy in macrophages [42]. K63-linked polyubiquitination has also been associated with the formation and autophagic degradation of protein inclusions [43]. Interestingly, the preferential binding of p62 to K63-linked chains constitute a further level of regulation during selective autophagy [44].

**Fig. 6:**
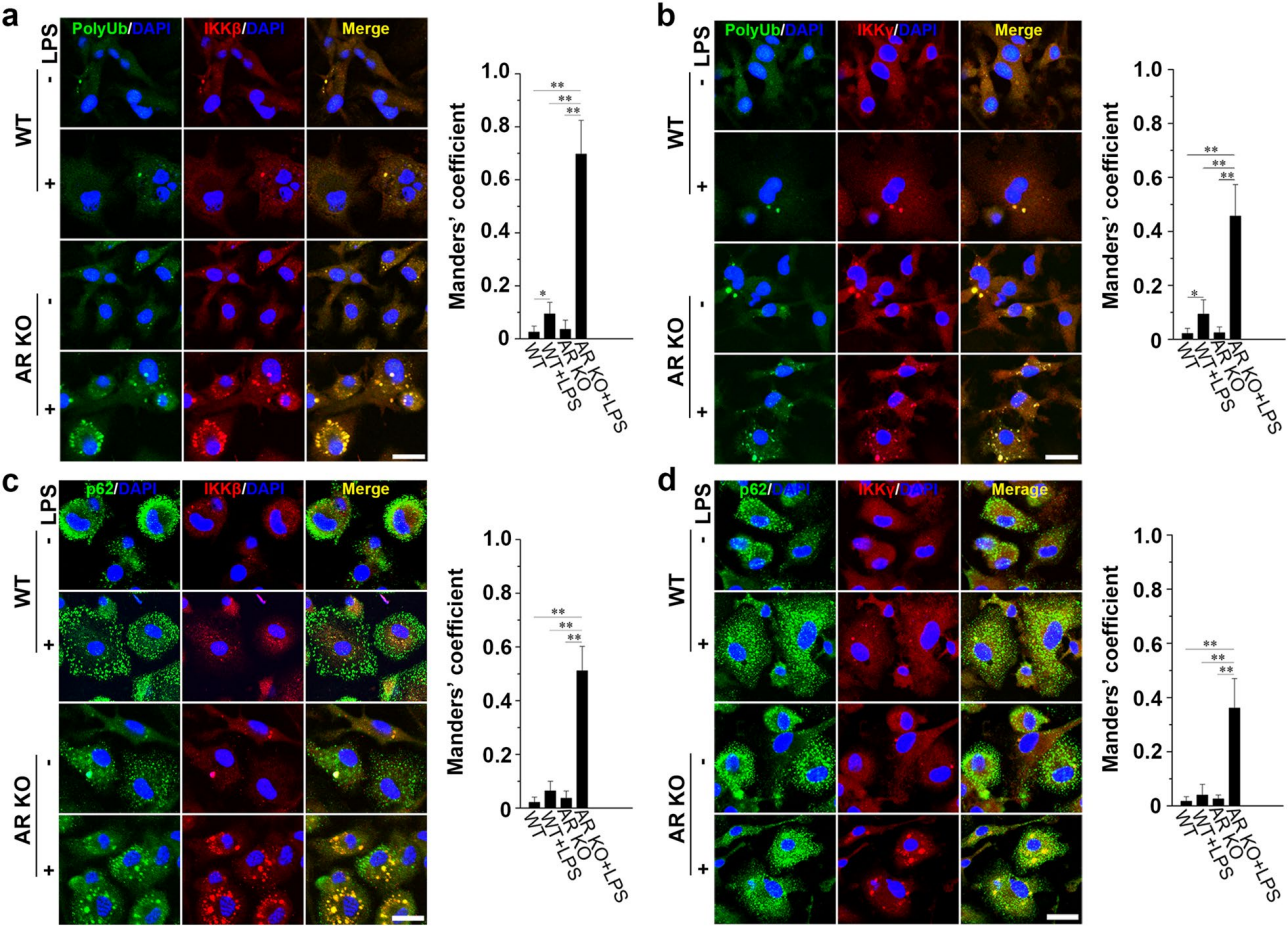
Connecting the IKK complex and autophagy pathway. Confocal microscopy images of BMMs from WT or AR KO mice treated with or without 500 ng/ml LPS for 1 h, and co-stained for **a** polyUb and IKKβ, **b** polyUb and IKKγ, **c** p62 and IKKβ, and **d** p62 and IKKγ. Right graphs represent the quantification of co-localization of signals using Manders’ overlap coefficient. Scale bars = 20 µm. Data are expressed as the mean ± SD from three independent experiments, *P < 0.05, **P < 0.01

Confocal microscopy of BMMs immunostained for PolyUb (linkage-specific K63) and IKKβ showed that in the absence of LPS stimulation, ubiquitin resided mainly in the cytosol where it appeared as small PolyUb^+^ dots, whereas IKKβ showed an occasional granular pattern (Fig. 6a). Treatment of BMMs with LPS for 1 h resulted in the formation of large IKKβ aggregates co-localized with PolyUb (Fig. 6a). Confocal microscopy results further suggested that in stimulated WT cells, only a small fraction of IKKβ was co-localized with PolyUb^+^; in contrast, the overlaps between IKKβ and PolyUb^+^ dots in LPS-treated AR KO cells were markedly increased (Fig. 6a). Similarly, we found that LPS stimulation induced the recruitment of IKKγ to PolyUb^+^ structures, and that the co-localization of IKKγ and PolyUb was markedly increased in AR KO cells (Fig. 6b).

Next, we treated primary macrophages with or without LPS and immunostained the cells for p62 and IKKβ. We noted that only a small fraction of p62 was associated with IKKβ in non-stimulated and LPS-treated WT cells, but this association was greatly increased in LPS-stimulated AR-deficient BMMs (Fig. 6c). Consistently, LPS-induced interactions between IKKγ and p62 were promoted by the knockout of AR in BMMs (Fig. 6d). These data indicated that IKKβ and IKKγ aggregates were polyubiquitinated and that they could be targeted by p62 to the autophagy pathway in LPS-induced AR-deficient macrophages.

## Discussion

Our results showed that AR deficiency led to the suppression of the M1 phenotype in macrophages, limiting its pro-inflammatory activity through the degradation of a key component of the IKK complex. Knockout of AR triggered autophagosome formation, and the exposure of AR KO macrophages to LPS markedly enhanced autophagosome biogenesis and maturation. Autophagy was able to capture and degrade IKKβ and IKKγ through polyubiquitination, leading to the recruitment of p62 and LC3. Autophagosome formation in the AR knockout background was functionally important; pharmacological blockade of autophagy pathway increased the protein levels of IKKβ and IKKγ and enhanced the production of iNOS. These data suggested a close relationship among AR, the M1 response, and autophagy.

There are two main NF-κB pathways in the cell. The noncanonical NF-κB signaling pathway depends on IKKα and plays a critical role in the development of lymphoid organs responsible for the generation of B and T lymphocytes [45]. Neither IKKβ nor IKKγ deficiency affects this pathway [45]. However, the canonical pathway, induced by most physiological NF-κB stimuli, depends on IKKβ and IKKγ. The activation of this pathway mainly leads to the phosphorylation of IκBα and the nuclear translocation of mostly p65-containing heterodimers [28, 46]. However, IKKγ deficiency appears to have a greater impact on the classical NF-κB signaling pathway than IKKβ deficiency [45]. It has been reported that cells lacking IKKγ have no detectable NF-κB response to almost any pro-inflammatory or immune regulatory stimuli [47]. Our results showed that the exposure of AR KO macrophages to LPS impaired both IKKβ and IKKγ. This may be attributed to the suppression of the TLR4/NF-κB signaling pathway, iNOS induction, and M1 polarization in AR-deficient BMMs.

We investigated the mechanism through which certain signals downregulate the IKKβ and IKKγ proteins without affecting their mRNA levels in AR KO macrophages after LPS treatment. We speculated that IKKβ and IKKγ protein levels were decreased at the post-transcriptional level in AR KO macrophages upon LPS stimulation. Previous studies showed that 4-hydroxynonenal (4-HNE) is an endogenous substrate for AR, with a higher affinity than glucose [48, 49]. Exogenous 4-HNE triggers autophagy, and deletion of AR increases 4-HNE accumulation and autophagy [17, 50]. We also found that the exposure of AR KO macrophages to LPS markedly increased the level of 4-HNE (Additional file 1: Fig. S1) and enhanced autophagosome formation. Furthermore, we investigated the relationship between the impairment of the IKK complex and autophagosome formation. We observed that a massive co-localization of IKKβ, IKKγ, LC3^+^ autophagic vacuoles, and LAMP1^+^ lysosomes happens in BMMs from AR KO mice after LPS stimulation. Furthermore, our finding that AR deficiency led to the polyubiquitination of IKKβ and IKKγ provides a possible answer. K63-ubiquitinated IKKβ and IKKγ can recruit p62, which can deliver them to the autophagy pathway through its LC3-binding domain. Conversely, pharmacological suppression of autophagy partially restored the protein levels of IKKβ and IKKγ as well as the induction of iNOS. Thus, manipulation of AR expression or activity may have a therapeutic potential by regulating macrophage polarization in inflammatory diseases.

## Supplementary Information

**Additional file 1: Figure S1.** LPS stimulation greatly alters the level of 4-HHE in AR KO macrophages.

## Data Availability

All data generated or analyzed during this study are included in this published article.
