# Correction to: Aldose reductase deficiency inhibits LPS-induced M1 response in macrophages by activating autophagy

**DOI:** 10.1186/s13578-021-00652-y

**Published:** 2021-07-18

**Authors:** Peng Cheng, Jianwei Xie, Zhiyong Liu, Jian Wang

**Affiliations:** 1 Department of Neurology, Second Naval Hospital of Southern Theater Command (425th Hospital of the People’s Liberation Army), Sanya, 572000 China; 2 Institute of Neurosciences, Fourth Military Medical University, Xi’an, 710032 China

## Correction to: Cell Biosci (2021) 11:61 https://doi.org/10.1186/s13578-021-00576-7

Following publication of the original article [1], the authors identified an error in the order of the affiliations. The correct order is given below:

Peng Cheng1, 2*, Jianwei Xie1, Zhiyong Liu1, Jian Wang2*

1Department of Neurology, Second Naval Hospital of Southern Theater Command (425th Hospital of the People’s Liberation Army), Sanya 572000, China.

2Institute of Neurosciences, Fourth Military Medical University, Xi’an 7110032, China.

The affiliations has been updated above and the original article [1] has been corrected.
