# Supplementary material for: Aldose reductase deficiency inhibits LPS-induced M1 response in macrophages by activating autophagy
Source: Cell Biosci. 2021 Mar 26;11:61. doi: 10.1186/s13578-021-00576-7 (PMC8004403; doi:10.1186/s13578-021-00576-7)
Supplement: Supplementary file 1 — Additional file 1: Figure S1. LPS stimulation greatly alters the level of 4-HHE in AR KO macrophages. [file 13578_2021_576_MOESM1_ESM.docx]

Additional File 1：

Figure. S1. LPS stimulation greatly alters the level of 4-HHE in AR KO macrophages.


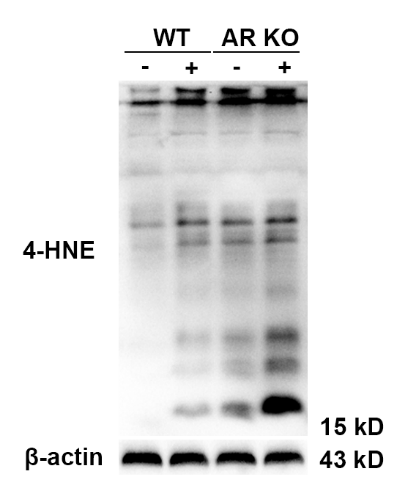


Figure. S1. LPS stimulation greatly alters the level of 4-HHE in AR KO macrophages. BMMs from WT or AR KO mice were treated with or without 500 ng/ml LPS for 16 h and then immunoblotted for 4-HNE.
